# Supplementary material for: DMRforPairs: identifying Differentially Methylated Regions between unique samples using array based methylation profiles
Source: BMC Bioinformatics. 2014 May 15;15:141. doi: 10.1186/1471-2105-15-141 (PMC4046028; doi:10.1186/1471-2105-15-141)
Supplement: Additional file 2 — DMRforPairs output for the comparison of A431-MCF7 and NA17018-NA17105. Please start from the HTML files in each folder. Available via the BMC Bioinformatics website. [file 1471-2105-15-141-S2.zip › 1394847754114233_MOESM2_ESM/A431_MCF7/figures/8335.pdf]

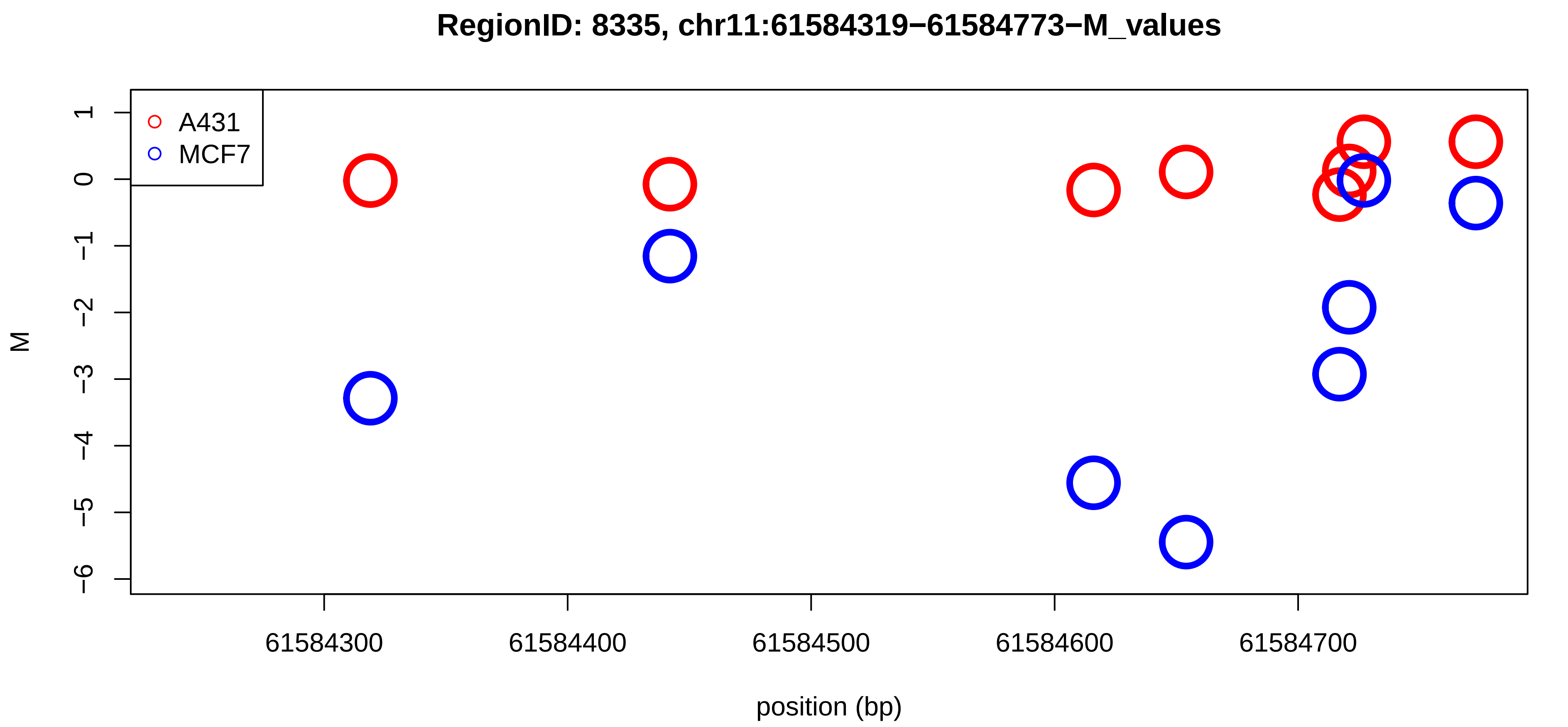
RegionID: 8335, chr11:61584319−61584773−M_values
A431
MCF7
M
1
0
−1
−2
−3
−4
−5
−6
61584300
61584400
61584500
61584600
61584700
position (bp)

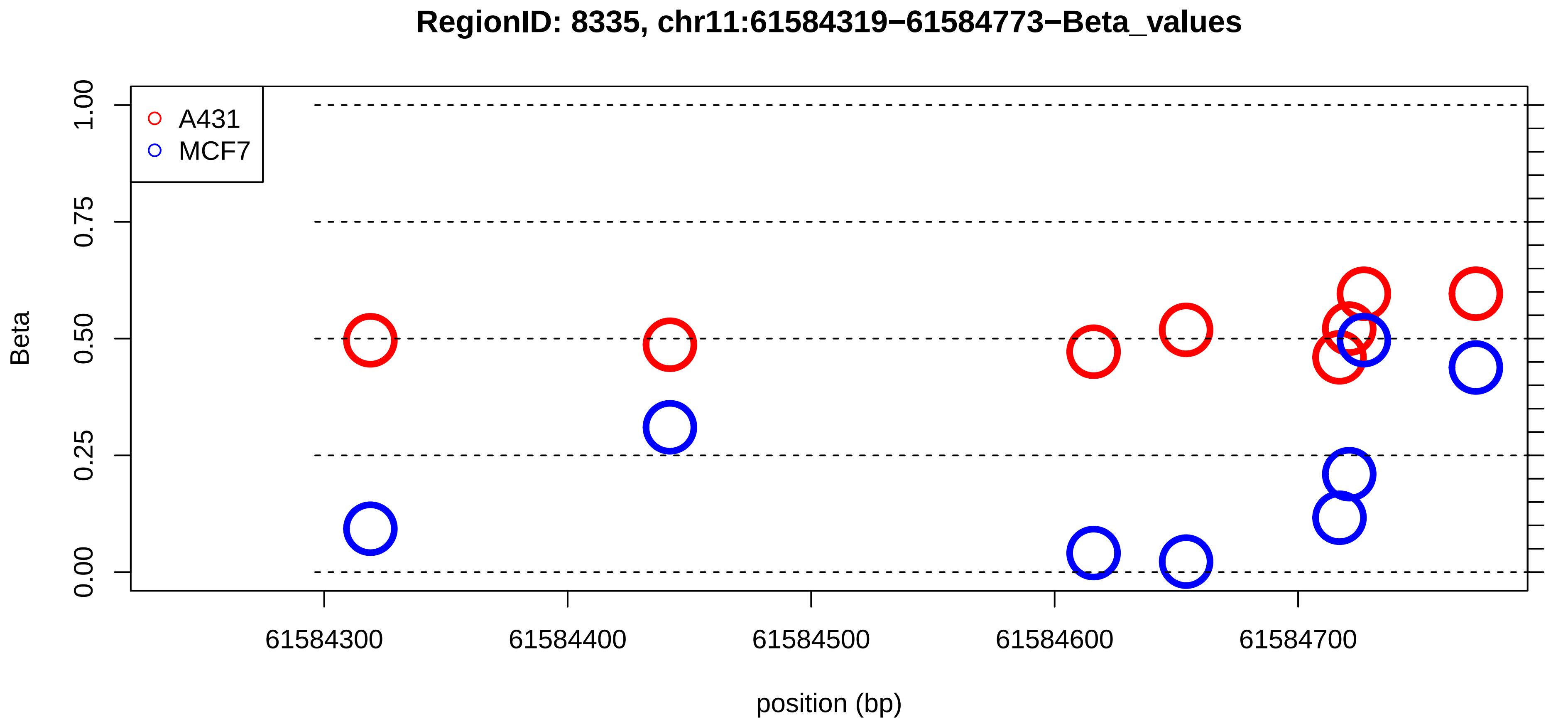
RegionID: 8335, chr11:61584319−61584773−Beta_values
A431
MCF7
Beta
1.00
0.75
0.50
0.25
0.00
61584300
61584400
61584500
61584600
61584700
position (bp)

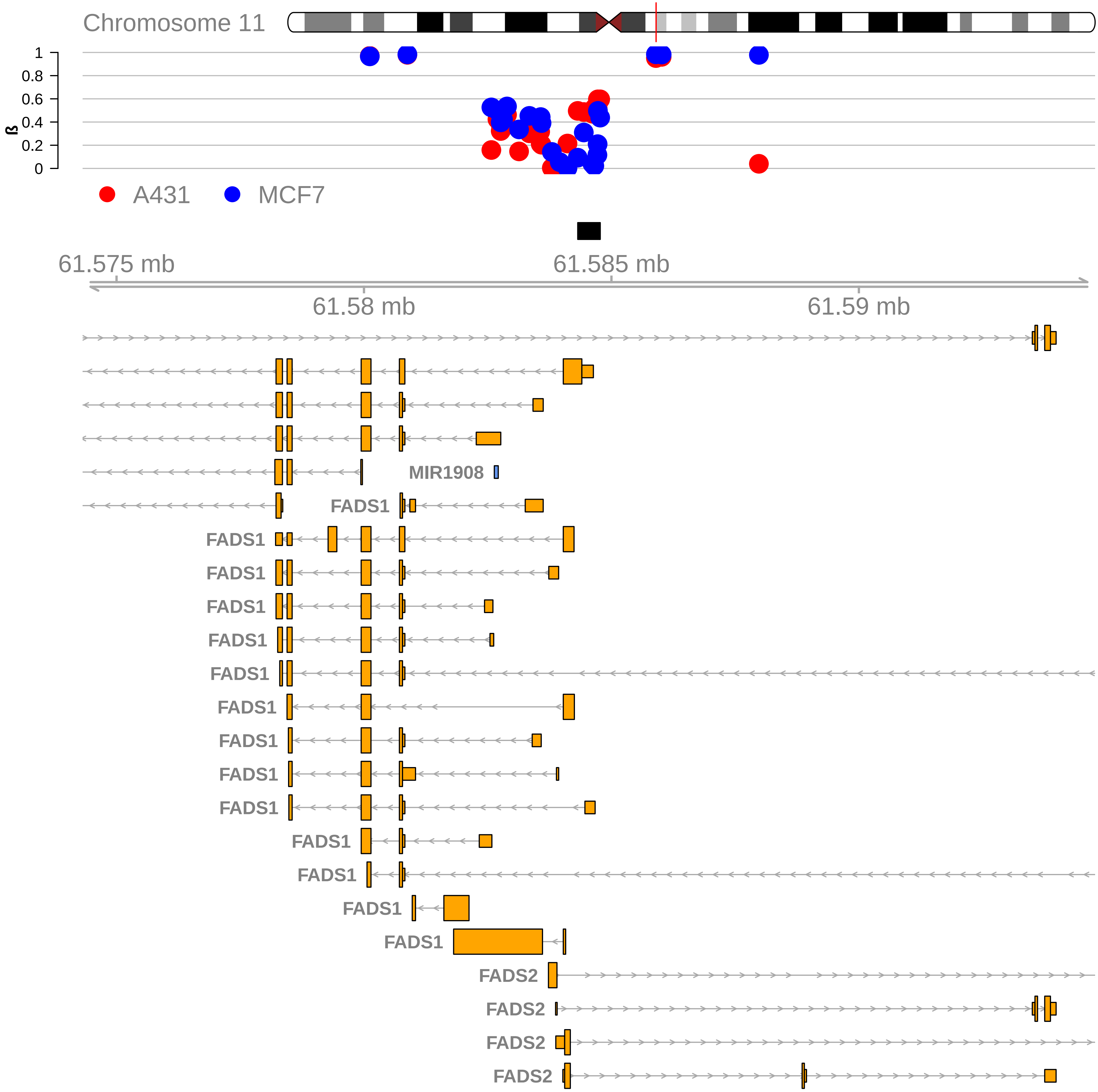

Chromosome 11
ß
1
0.8
0.6
0.4
0.2
0
A431
MCF7
61.575 mb
61.585 mb
61.58 mb
61.59 mb
MIR1908
FADS1
FADS1
FADS1
FADS1
FADS1
FADS1
FADS1
FADS1
FADS1
FADS1
FADS1
FADS1
FADS1
FADS1
FADS2
FADS2
FADS2
FADS2
